# Characterization of JG024, a pseudomonas aeruginosa PB1-like broad host range phage under simulated infection conditions

**DOI:** 10.1186/1471-2180-10-301

**Published:** 2010-11-26

**Authors:** Julia Garbe, Andrea Wesche, Boyke Bunk, Marlon Kazmierczak, Katherina Selezska, Christine Rohde, Johannes Sikorski, Manfred Rohde, Dieter Jahn, Max Schobert

**Affiliations:** 1 Institute of Microbiology, Technische Universität Braunschweig, Spielmannstr. 7, 38106 Braunschweig, Germany; 2 DSMZ, German Collection of Microorganisms and Cell Cultures, Inhoffenstr. 7B, 38124 Braunschweig, Germany; 3 HZI, Helmholtz Centre for Infection Research, Inhoffenstr. 7, 38124 Braunschweig, Germany

## Abstract

**Background:**

*Pseudomonas aeruginosa *causes lung infections in patients suffering from the genetic disorder Cystic Fibrosis (CF). Once a chronic lung infection is established, *P. aeruginosa *cannot be eradicated by antibiotic treatment. Phage therapy is an alternative to treat these chronic *P. aeruginosa *infections. However, little is known about the factors which influence phage infection of *P. aeruginosa *under infection conditions and suitable broad host range phages.

**Results:**

We isolated and characterized a phage, named JG024, which infects a broad range of clinical and environmental *P. aeruginosa *strains. Sequencing of the phage genome revealed that the phage JG024 is highly related to the ubiquitous and conserved PB1-like phages. The receptor of phage JG024 was determined as lipopolysaccharide. We used an artificial sputum medium to study phage infection under conditions similar to a chronic lung infection. Alginate production was identified as a factor reducing phage infectivity.

**Conclusions:**

Phage JG024 is a suitable broad host range phage which could be used in phage therapy. Phage infection experiments under simulated chronic lung infection conditions showed that alginate production reduces phage infection efficiency.

## Background

*Pseudomonas aeruginosa *is well known as an opportunistic human pathogen characterized by a high intrinsic antibiotic tolerance [1,2]. In humans, *P. aeruginosa *can cause urinary tract, respiratory tract, and burn wound infections [3-5]. Respiratory tract infections caused by *P. aeruginosa *are dreaded in patients suffering from the genetic disorder Cystic Fibrosis (CF) [2,6,7]. CF patients exhibit an increased mucus production in the lung [8]. Bacteria like *P. aeruginosa *are able to colonize this mucus and cause chronic infections, which cannot be eradicated by antibiotic treatment [4]. Several hypothesis exist explaining the observed high antibiotic tolerance of *P. aeruginosa *in the CF-lung, which is caused by special growth conditions. These include growth as biofilm-like microcolonies, which have been shown to increase antibiotic tolerance up to 1000-fold [9,10]. A couple of *in vitro *model systems have been described to simulate a CF lung infection caused by *P. aeruginosa *[11-13]. The artificial sputum medium is a complex medium based on components measured in the CF sputum [12]. It mimics the CF-lung environment during infection and causes typical *P. aeruginosa *phenotypes as mucoidy and microcolony formation [12]. Since eradication of chronic *P. aeruginosa *infections by antibiotics fails, phage therapy is a possibility to treat bacterial infections. Advantages over antibiotics are the specificity of phages and that phages can be isolated and investigated rapidly [14]. For this reason, several suitable *P. aeruginosa *broad host range phages have been characterized. The *Pseudomonas *infecting PB1-like phages are widespread in nature and possess highly conserved genomes. Comparative genome analysis of five PB1-like (PB1, SN, 14-1, LMA2 and LBL3) phages was recently published and is the first genome report for these phages [15]. PB1-like phages belong to the *Myoviridae *phage family and the genome sizes vary between 64,427 and 66,530 bp. The genomes encode for 88 (LBL3) to 95 proteins (LMA2) [15]. More than 42 phages have been reported to be PB1-like. These results are mainly based on DNA hybridization and morphological studies [15,16]. More recently, PB1-like phages as phage 14-1 have been reported as part of a well defined phage cocktail to treat *P. aeruginosa *burn wound infections [17]. The application of phages as a therapeutical agent requires an in depth understanding of the phage biology [18]. Moreover, phages which multiply well under *in vitro *conditions can fail to replicate during treatment *in vivo *[19]. Therefore, phages and especially the ability of the phage to infect the host *in vivo *should be investigated carefully prior to use. Here we describe the in depth characterization of a broad host range PB1-like phage with a slight prevalence to clinical isolates. We used an artificial sputum medium to simulate the conditions in the CF lung and investigated the ability of phage JG024 to infect *P. aeruginosa *and multiply under these conditions.

## Results and Discussion

### Isolation and host range of phage JG024

Phages were isolated from sewage as described in Methods. We isolated 59 *P. aeruginosa *specific phages and used an initial set of 5 different *P. aeruginosa *strains as the laboratory strains PAO1, PA14 as well as three clinical isolates (BT2, PACF15 and MH19, Table 1) to test the host range. One phage, which was named JG024, was able to conduct clear lysis on this set of bacterial strains. To determine the host range of JG024 in more detail, we used 19 clinical isolates from CF patients and from urinary tract infections as well as a collection of 100 environmental strains (Table 1). JG024 is able to infect 84% of all tested clinical isolates. Furthermore, JG024 is even capable of infecting a *P. aeruginosa mucA *mutant and the clinical isolate BT73, which both showed the same mucoid phenotype. *mucA *mutants produce large amounts of the exopolysaccharide alginate and mutations in *mucA *are critical for the conversion of non-mucoid to mucoid *P. aeruginosa *variants in the lung of CF patients [20,21]. Additionally, we determined the host range of the phage JG024 with a collection of 100 *P. aeruginosa *environmental strains isolated from different rivers (Oker, Aller, Weser) in Lower Saxony, Germany. The results showed that JG024 was able to infect 50% of the strains. Interestingly, phage JG024 showed a clear lysis for only 45% of the 50 lysed environmental isolates but was able to conduct clear lysis on 68% of the 19 lysed clinical isolates.

**Table 1:** Strains and phages used in this study.

Bacterial strain or phage	Phenotype or genotype	Reference
PAO1	wild type	[48]

PA14	wild type	[49]

FRD1	mucoid CF isolate	[34]

PAO1 Δ*mucA*	PAO1 *mucA*::*aacC1-gfp *Gm^R^	Sabrina Thoma, this laboratory, unpublished

PAO1 Δ*pilA*	*pilA *inactivated by allelic displacement; tagged with eGFP, Tc^R^, Gm^R^	[50]

PAO1 Δ*fliM*	*fliM *inactivated by allelic displacement; tagged with eGFP, Tc^R^, Gm^R^	[50]

PAO1 Δ*algC*	PAO1 *algC *::*aacC1-gfp *Gm^R^	Julia Garbe, this laboratory, unpublished

BT2, BT72, BT73, RN3, RN43, RN45, NN84	clinical CF isolates	Medical Highschool Hannover, Germany

PACF15, PACF21, PAKL1, PAKL4, PACF60, PACF61, PACF62, PACF63	clinical CF isolate	Gerd Döring, Tübingen, Germany

Nr. 18, 19, 26, 29	urinary tract infection isolate	Michael Hogardt, München, Germany

Environmental strains		Katherina Selezska, HZI Braunschweig, Germany

JG024	wild type PAO1 LPS specific lytic bacteriophage	this study

### Family affiliation of JG024

To determine family affiliation of phage JG024, we determined the nature of the nucleic acids and the morphology of the phage to assign the family by comparison [22]. Nucleic acids were isolated as described in Methods and identified as dsDNA due to its sensitivity to restriction endonucleases like *Sac*II, which cut only dsDNA. *Sac*II produced distinct fragments of approximately 30 kb, 25 kb and 8 kb (data not shown). Computational analysis of the *Sac*II restriction sites in the sequenced genome (see below) revealed slightly different fragment sizes of 28,348 kb and 21,719 kb, respectively as well as two fragments with a size of 8,49 kb and 7.718 kb, which we observed as one 8 kb fragment.

**Figure 1:**
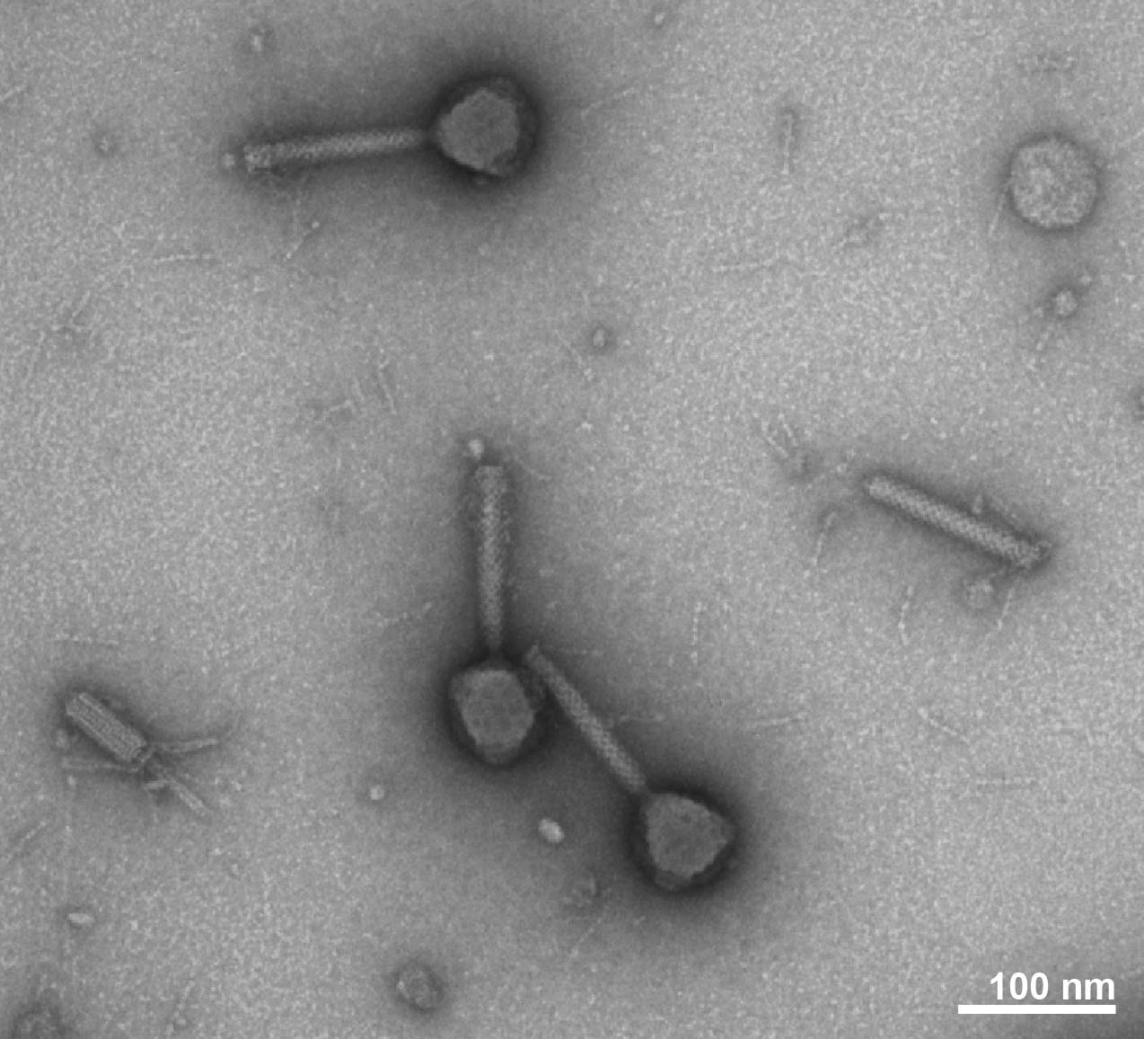
**Morphology of JG024**. Electron microscopic image of negatively stained JG024 phages, which exhibit a contractile tail with a length of 130 nm. The icosahedral head of JG024 has a length of 80 nm and a width of 75 nm.

Electron microscopy (Figure 1) shows an icosahedral head with a length of 80 nm and a width of 75 nm. The contractile tail, which consists of a neck, a contractile sheath and a central tube has a length of approximately 130 nm. Due to these morphological results and in accordance with the presence of dsDNA, the phage JG024 is grouped to the family *Myoviridae*. This family is a member of the order *Caudovirales *which contains exclusively tailed phages also from the families *Siphoviridae *and *Podoviridae*.

### Receptor of phage JG024

We used different *P. aeruginosa *mutants to identify the receptor of phage JG024 as outlined by others [23]. Aflagella mutant (Δ*fliM*), a pili mutant (Δ*pilA*) and an LPS mutant (Δ*algC*) were infected with the phage JG024. After incubation, lysis was investigated on bacterial lawns (data not shown). JG024 lyses the pili- and the flagella mutant but not the *P. aeruginosa ΔalgC *mutant. The *algC *gene encodes an enzyme with phosphoglucomutase and phosphomannomutase activity. A *P. aeruginosa *Δ*algC *mutant produces a truncated LPS core and lacks common antigen suggesting that these structures might constitute the host receptor for JG024 attachment [24,25].

### Growth characteristics

**Figure 2:**
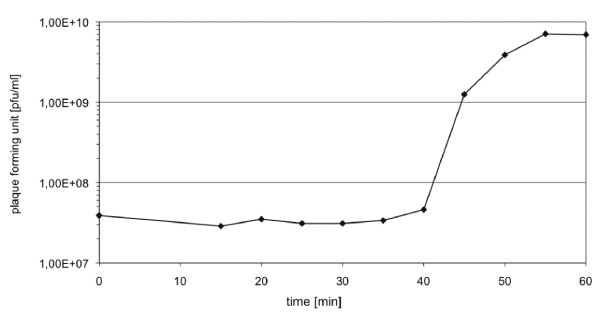
**Growth characteristics of JG024**. One step growth curve of phage JG024. A representative growth experiment of three independent experiments is shown. The latent phase of JG024 takes approximately 50 min and the phage is able to produce about 180 phage progeny per infected cell.

To investigate growth parameters like the latent phase and the burst size of the phage JG024, we performed single step growth curves as described in Methods, Figure 2. Phage JG024 has an estimated latent phase of 50 min. The burst size, which describes the mean number of phages liberated per bacterial cell was determined as 180 phages per infected cell.

### JG024 is a PB1-like phage

Phage JG024 DNA was sequenced and assembled at McGill University as described in Methods. The genome size of phage JG024 is 66,275 bp and has a GC content of 55.62%. Genome comparison using the blastx tool revealed that phage JG024 is highly related to the widespread and conserved PB1-like viruses [15,26]. It was recently reported that these phages show a high sequence similarity and limited horizontal gene transfer [15]. The general characteristics as well as the similarity to phage JG024 are shown in Table 2. The overall nucleotide similarity to PB1-like phages varies between 86% to phage PB1 and 95% to the phages SN and 14-1 (Table 2). We also compared the JG024 genome sequence with PB1 and SN using Mauve [27] and detected only few insertions or deletions, Additional file 1 Figure S1. Due to the high sequence similarity, the broad host range characteristic as well as the morphology, we conclude that phage JG024 belongs to the PB1-like phages. In accordance with our findings, PB1-like phages also have been shown to use LPS as receptor [28]. Since the sampling location of JG024 in Lower Saxony, Germany is different to all other PB1-like phages, it underscores the broad environmental distribution of this phage group probably due to the broad host range [15].

**Table 2:** Comparison of the JG024 genome to the genomes of PB1-like phages 15.

Phage	Genome size (bp)	GC content (%)	Predicted ORFs	unique ORFs	DNA identity (%) to JG024
JG024	66,275	55.62	94	1	100

PB1	65,764	55.5	93	-	86

F8	66,015	55.6	93	1	87

SN	66,390	55.6	92	2	95

14-1	66,238	55.6	90	-	95

LMA2	66,530	55.5	95	2	93

LBL3	64,427	55.5	88	2	92

### Features of the JG024 genome

**Figure 3:**
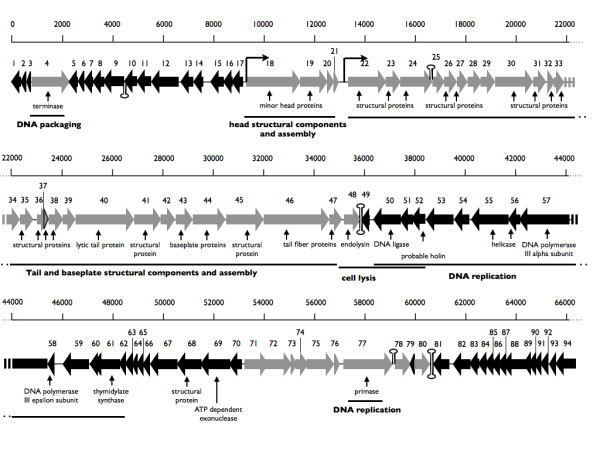
**Genome of JG024**. Schematic representation of the JG024 genome with its assumed ORFs and some functional assignments. The arrowheads point in the direction of transcription. Detected putative sigma70-promoters as well as potential terminator hairpin structures are indicated. The complete genome is submitted with GenBank (NCBI, accession number: GU815091).

The schematic representation of the genome, with its assumed ORFs, some functional assignments and overall genetic organization is depicted in Figure 3. The genome of JG024 is compact organized with only 7.1% intergenic space. No genes encoding for tRNAs were found in the genome of JG024 using the program RNAscan-SE 1.21 [29]. Interestingly, the GC content of phage JG024 differs from its host (55.62% to 68%). Comparison of the codon usage of JG024 with its host *P. aeruginosa *showed that the phage shares the same dominant codons for each amino acid except for valin, serin and glutamate. To test if the genome of phage JG024 is linear or circular, we used a method described previously [30]. A linear genome of phage JG024 was identified by treatment with exonuclease *Bal*31 which degrades only double-stranded linear DNA from both ends simultaneously (data not shown). However, we did not identify the exact genome ends. This would indicate that the genome of phage JG024 is circular permuted in contradiction to the PB1 phages, which have been reported to have non-permuted linear genomes [15]. Since the terminase protein of JG024 is highly (up to 99.6%) identical to that of the PB1 phages, we assume phage JG024 to have a non-permuted linear genome.

Since these phages share a high sequence similarity a comparative ORF prediction was possible. First, the heuristic approach of GeneMark was used to identify genes in small genomes under 100 kb [31]. With this approach a total of 84 putative ORFs were identified. In a second approach we used the NCBI ORF Finder program coupled with the program blastp and compared the translated proteins with the proteins of the PB1-like phages [26,32]. Combination of the results of both approaches revealed a total of 94 predicted ORFs as well as one unique ORF in phage JG024.

No RNA polymerase was detected suggesting that this phage uses the host transcriptional machinery, as it was also suggested for the PB1-like family of phages.

We detected a putative structural gene cluster which contains genes encoding for putative head structure proteins (ORF 18 and 19) as well as for tail and baseplate proteins (ORF 22-47). Moreover, ORF 40 was designated as a lytic tail protein. It was shown for the phages 14-1 and LBL3 that this protein has a transglycosylase domain with a N-acetyl-D-glucosamine binding site, which shows a specific degradation of peptidoglycan [15]. ORF 48 encodes a putative endolysin with a high similarity to the endolysin of phage LMA2 (98.6%) and belongs to a lysozyme-like superfamily. A putative holin may be encoded by ORF 52, which shares a 100% identity to ORF 50 of phage F8 and to ORF 51 of phage 14-1. It was suggested that these ORFs encode probable holins since they are located near the endolysin gene and they encode a small protein (201 aa) containing three transmembrane domains [15].

Additionally, a complete DNA replication machinery was detected suggesting that the DNA replication is host independent as described for the PB1-like phages. The respective gene cluster contains a DNA ligase (ORF 50), a helicase (ORF 55 and 56), a DNA polymerase III (ORF 57 and 58), as well as a thymidylate synthase (ORF 61). A putative primase was also found but is not included in this gene cluster (ORF 77), as shown for the other PB1-like phages [15]. Also, differences between the PB1-like phages and JG024 were found. Phage 14-1 (ORF 71) and phage LBL3 (ORF 68) encode a hypothetical protein with a size of 434 aa. Interestingly, this protein is encoded by two ORFs in phage JG024 designated ORF 72 (362 aa) and 73 (60 aa). The two ORFs are separated by only 116 bp. Moreover, ORF 79 is a small predicted gene with a size of 132 bp and encodes for a unique protein in phage JG024. This ORF was identified by two programs, GeneMark and ORF Finder, independently. No functional indication could be pointed out since there are no similarities to other proteins in the databases and no conserved domains have been detected in ORF 79. We also searched the genome of phage JG024 for promoters, terminators and regulatory elements, see Methods. The PB1 phages do not contain a phage RNA-polymerase and depend on the transcriptional machinery of the host bacterium. Putative sigma 70-promoter regions have been predicted in PB1 phages [15]. We detected two putative sigma 70-promoter regions in the 5'-region of ORF 18 and ORF 22, similar to the position of the putative promoters found in PB1 phages, see Table 3 and Figure 3[15]. In addition, we also determined the location of six rho-independent transcriptional terminators and checked if their position is conserved to the other PB1 like phages, Table 3 and Figure 3. Moreover, we searched for additional conserved motifs in intergenic regions using MEME and detected AT-rich boxes and additional conserved motifs in intergenic regions. However, the function of the motifs is unclear, their position indicates a possible function as a recognition sequence for a phage sigma factor as suggested earlier [15].

**Table 3:** Potential regulatory elements and intergenic motifs of the JG024 genome.

Position	ORF	Sequence	Orientation	Score	dG (kcal mol^-1^)
putative *σ*70-dependent promoter elements:					

9286..9336	ORF18	ATGTTTGAATCTCT**TTTGAA**CGT TTGATGTTTCCCC**TATAA**TAAGC GCACA	Forward	1.22	

13050..13100	ORF22	TCATCTATAAGTA**ACGTTAT**AAC ATAACGTCAATTTA**TATGCT**CTA GACGT	forward	1.19	

putative rho-independent terminator elements:					

2313..2343	ORF 10	**AAGCCCGGA**GCGA**TCCGGGCTT**T TCTGTGTT	reverse		-17.5

16623..16644	ORF24	**GGCCGG**GTT**TCCGGCCTT**TGTT	forward		-12.3

35910..35942	ORF48	**AAAAGGCCGCT**TATTC**AGCGGCC TTTT**TGCTTT	forward		-18.3

35931..35900	ORF49	**AAAAGGCCGCT**GAATA**AGCGGCC TTTT**CTTTT	reverse		-18.3

59033..59059	ORF77	**AGGCCGCC**TTCG**GGCGGTCT**TTT CTTT	forward		-14.7

60667..60706	ORF80	**AAAGCCCCGG**AC**TCT**AGTTC**AGA A**T**CCGGGGCTTT**CTTTT	forward		-23.8

60700..60657	ORF81	**AAAGCCCCGG**AT**TCT**GAACT**AGA G**T**CCGGGGCTTT**GTCGCTTCT	reverse		-23.8

Position and orientation of putative sigma 70 promoters and putative rho-independent terminator regions. The putative promoters were identified using SAK and Virtual Footprint as described in Methods. "Orf" indicates the Orf in the 3'-region of the putative promoter. Bold letters of the promoter sequences indicate -35 and -10 regions. The putative terminator regions were identified using the programs TransTerm and FindTerm as described in Methods. The indicated Orf is the respective Orf in the 5'-region of the putative rho-independent terminator.

### ASM infection assay

**Figure 4:**
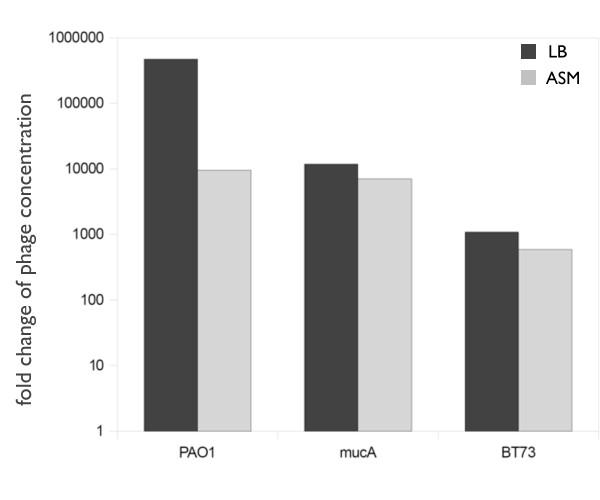
**Infection assay of JG024 in ASM medium**. Phage growth during infection assay in LB medium (dark grey bars) and ASM medium (light grey bars). Changes in phage concentration are described as n-fold.

Since phage JG024 is able to infect 84% of the tested clinical isolates *in vitro *we were interested if this phage is able to infect *P. aeruginosa *under simulated CF lung conditions. An artificial sputum medium (ASM) was used to mimic the CF lung environment. Growth in ASM leads to formation of typical biofilm-like microcolonies of *P. aeruginosa *and supports other phenotypic changes observed under chronic infection conditions [12]. At first, we tested the ability of phage JG024 to lyse the non-mucoid wild type strain *P. aeruginosa *PAO1 in ASM compared to LB medium. As described in Methods, we monitored phage particles and noted an increase of phage particles by a factor of nearly 500 000 in LB and in ASM by a factor of 10 000 (Figure 4). This indicates cell lysis by phage JG024 under these non ideal conditions (note that PAO1 was in stationary phase prior to infection to allow comparison to ASM). When we monitored infection of *P. aeruginosa *PAO1 in ASM we noticed a 50-fold lower concentration of phage particles. This indicates a reduced efficiency of phage infection by JG024 under simulated chronic infection using the artificial sputum medium. In parallel we tested a *P. aeruginosa *CF-isolate, strain BT73, for susceptibility to phage infection in LB and ASM. Unexpectedly, we noticed only a 1.9-fold lower phage number in ASM compared to LB (Figure 4). We noticed that phage JG024 was less effective against the CF isolate under both conditions, since approximately tenfold less phage particles were produced under both conditions compared to PAO1. However, while strain BT73 is less susceptible to phage lysis, the efficiency does not decrease dramatically under ASM growth conditions.

In contrast to the *P. aeruginosa *PAO1 strain the CF-isolate BT73 is mucoid and secretes the exopolysaccharide alginate. We wondered if alginate overproduction could explain the observed results. It was recently published that even non-mucoid strains like the wild type PAO1 express the exopolysaccharide alginate in response to oxygen-limiting conditions [33]. We also observed that cultures of PAO1 in ASM, which mimics the CF lung, were highly viscous compared to the cultures in LB medium, suggesting a high production of alginate by the wild type PAO1 in this medium. If alginate is the factor in our experimental setup which decreases phage infection efficiency, a mucoid variant of strain PAO1 should show a similar result as the clinical isolate BT73. Therefore, we repeated the phage infection experiments in LB and ASM with a *P. aeruginosa mucA *mutant strain. We observed again only a 1.6-fold decrease in ASM and an overall approximately tenfold reduction in phage particles when compared with *P. aeruginosa *PAO1 (Figure 4). These results are in agreement with our hypothesis that alginate overproduction reduces phage infection efficiency. Moreover, they point to alginate as the dominant factor for the decrease in phage infection efficiency in ASM. To verify this result, we performed the same experiment with *P. aeruginosa *PAO1 in LB medium and increasing alginate concentrations. We chose alginate concentrations of 50, 100, 200, 500 *μ*g/ml up to 1000 *μ*g/ml, since non-mucoid *P. aeruginosa *strains have been reported to produce 50-200 *μ*g/ml alginate, while mucoid isolates produce up to 1000 *μ*g/ml alginate [34-36]. In accordance with our hypothesis, the presence of alginate reduced phage multiplication in our test assay. A concentration of 50 to 200 *μ*g/ml alginate resulted in an almost 20-fold reduction of phage particles compared to LB medium alone in accordance with the 50-fold reduction of phage particles observed in ASM compared to LB. This effect is even more obvious with alginate concentations of 500 and 1000 *μ*g/ml, where we observed a reduction of phage particles by a factor of 130 and almost 2800, respectively.

## Conclusions

We could show that the phage JG024 belongs to the PB1-like phages and shares several characteristic features of this group. These phages are widespread in nature and very successful. A new member of this group, phage JG024, was isolated and characterized. General growth characteristics as well as the genome were investigated, showing that JG024 is able to pass one infection cycle in approximately 50 min. Genome analysis revealed the strong relatedness to the PB1-like phages. Moreover, we could show that JG024 has broad spectrum activity with a prevalence to clinical isolates. Also, infection of the host *P. aeruginosa *was even possible under challenging conditions like the ASM medium which mimics the CF lung. High viscosity and microcolony growth of the host were only small obstacles for JG024 to infect and multiply under these conditions. These results show that this group of bacteria could be an important contribution to phage therapy. Moreover, we established a method to investigate the possibility of a phage to lyse bacteria under infection conditions prior to use for phage therapy *in vivo*.

## Methods

### Bacterial strains and growth conditions

Table 1 shows the genotype and phenotypes of the bacteria and phage JG024 used in this study. The 100 environmental *Pseudomonas aeruginosa *strains used in this study origin from a comprehensive screen of approx. 400 environmental river strains. These were genetically characterized using the ArrayTube hybridization chip [37]. The 100 strains used here are all different in their core genomic SNP pattern and were chosen such to represent the entire population genetic diversity currently known for *P. aeruginosa*. Details of the comprehensive screen will be published elsewhere. *P. aeruginosa *strains were routinely propagated in Luria Bertani (LB) broth medium aerobically at 37°C. The composition of the artificial sputum medium (ASM) is described elsewhere [12].

### Phage Isolation

Phages were isolated from sewage following a simple enrichment procedure. Samples from a sewage plant Steinhof in Braunschweig, Germany were centrifuged for 5 min at 4100 × g (Biofuge fresco). Ten ml of the supernatant were mixed with 5 ml of a *P. aeruginosa *overnight culture and incubated in 50 ml LB broth at room temperature. After an incubation of 48 h, the cells were sedimented by centrifugation at 4100 × g (Biofuge fresco) for 10 min and the supernatant was transferred to a clean tube. To kill remaining bacteria, several drops of chloroform were added to the supernatant and the emulsion was mixed for 30 s. To separate the phages, appropriate dilutions of the phage lysate were spotted onto bacterial lawns of top-agar plates. Top-agar plates were produced by adding approximately 5*10^8 ^cells/ml of *P. aeruginosa *from an overnight LB broth to 3.5 ml of LB top-agar (0.75%). The inoculated top-agar was overlaid on an LB agar plate and allowed to solidify. After incubation at 37°C for 10 to 16 h zones of lysis were monitored. Single plaques, derived from a single phage, were separated by stinging with a pipette tip into the plaque followed by resuspending the phages in SM buffer (100 mM NaCl, 8 mM MgSO_4_, 50 mM Tris-HCl, pH 7.5). The resulting phage lysate was stored at 4°C.

### Electron microscopy

The morphology of the phages was detected by negative staining with uranyl acetate and transmission electron microscopy. Phages were allowed to absorbe onto a thin carbon film, prepared on mica, from a liquid sample for different time points, washed in TE buffer (10 mM TRIS, 2 mM EDTA, pH 6.9) and distilled water. Phages were negatively stained by floating the carbon film for approx. 15 sec on a drop of 2% aqueous uranyl acetate. Then, the carbon film was picked up with copper grids (300 mesh), blotted semi-dry with filter paper and was subsequently air dried. Samples were examined in a Zeiss EM910 transmission electron microsope at an acceleration voltage of 80 kV and at calibrated magnifications. Images were recorded digitally with a Slow-Scan CCD-Camera (ProScan, 1024 × 1024, Scheuring, Germany) with ITEM-Software (Olympus Soft Imaging Solutions, Münster, Germany). Brightness and contrast were adjusted with Adobe Photoshop CS3.

### Phage host range spectrum and detection of host receptor

To determine the phage host range, top-agar plates with the potential host lawn were prepared. Top-agar plates were produced by adding approximately 5*10^8 ^cells/ml of *P. aeruginosa *from an overnight LB broth to 3.5 ml of LB top agar (0.75%). Ten *μ*l of a phage stock solution were spotted on the top-agar plate and incubated at 37°C for 12 to 16 h. After incubation, the appearance of the lysis zones at the site where the phage suspension was added, was examined. Each phage was tested against each bacterial strain in triplicate in independent experiments. The lysis was categorized as clear (+), turbid (0) and no reaction (-) as described [38]. For detection of the phage receptor molecule, we used a *P. aeruginosa *flagella mutant (Δ*fliM*), a pili mutant (Δ*pilA*) and an LPS mutant (Δ*algC*), which were infected with the phage JG024 as described above. The strains for the receptor identification are derived from a PAO1 wildtype and therefore belong to the same serotype as PAO1, namely serotype O5 [39]. An effect on the efficiency of plating was not observed for the strains with intact LPS.

### Phage growth characteristics

To determine phage growth characteristics like burst size and duration of the infection cycle, single step growth experiments were performed as previously described with some modifications [40,41]. *P. aeruginosa *was grown aerobically in 10 ml LB medium until exponential growth phase. After the bacteria reached an OD578 of 0.3, an aliquot containing 5*10^8 ^phages was added to the culture which corresponds to a multiplicity of infection (MOI) of 0.16. Therefore, it is likely that a cell is infected by only one phage and that the amount of infected bacteria is equal to the amount of the initial phage concentration. After addition of the phages, one aliquot was immediately used for determination of the phage titer. Then, phages were allowed to adsorb for 15 min. Afterwards, cultures were diluted in LB 10^4^-, 10^5^-, 10^6^- and 10^7 ^-fold and incubated at 37°C for 60 min. Samples for phage enumeration were taken aseptically at different time points after infection. The burst size was determined as: (phage titer at the end of the single step growth curve at time point 55 min minus phage titer at time point 20 min) divided by phage titer at time point 20 min. The latent phase was estimated at the midpoint of the exponential phase of a one step growth experiment [40,41].

### Sequencing, analysis and annotation of phage genomes

To isolate phage DNA, phages were propagated in top-agar plates as described above. After growth at 37°C the plates were overlayed with 10 ml SM buffer and incubated with shaking at 4°C for 4 h. The supernatant was sterile filtrated (0.22 *μ*m) and stored at 4°C. Phage DNA was isolated using the Qiagen Lambda Kit according to manufacturer's instructions. Ten ml phage lysate with a titer of at least 1*10^10 ^phages/ml were used to isolate up to 1 *μ*g/*μ*l pure phage DNA. Digestion with restriction endonucleases was done following the protocols of the manufacturer. Whole genome sequencing of the phage JG024 was done at the McGill University and Génome Québec Innovation Centre (Montréal, QC, Canada) using the Genome Sequencer FLX and 454 Technology. A total of 66,684 reads with an average length of 344 bases was assembled to one single contig with a 300-fold coverage. The annotation of the unknown phage genes was done by using the software GeneMark.HMM [31]. The Heuristic approach of GeneMark was used to identify genes in small genomes under 100 kb. The identified genes were compared with the NCBI ORF Finder [32]. Nucleotide sequences were scanned for homologues using the Basic Alignment Search Tool (blastx) [26]. To search for tRNA genes in the phage sequences the internet tool tRNAscan-SE 1.21 was used [29]. Sequence comparison was conducted using ClustalW2 online analysis tool [42]. Investigation of the codon usage was performed using a software tool based on JCat [43]. The genome sequence as well as the annotation is deposited with the GenBank (National Center for Biotechnology Information) using the following accession number: GU815091.

### Identification of promoter regions, terminator structures and other motifs

The genome of phage JG024 was scanned for the presence of sigma 70-dependent promoter regions using the web service SAK [44]. Putative promoter regions with a score above 1 were scanned for the presence of conserved -10 and -35 regions using the Virtual Footprint software [45]. Two promoter regions were identified in this way. Rho-independent terminator structures were identified using the TransTerm [46] and FindTerm (Softberry, Inc.) software tools. The program MEME was used for identification of conserved intergenic motifs in phage JG024 [47].

### ASM infection assay

Phage susceptibility of *P. aeruginosa *in ASM medium was tested in 24 well plates. 1 ml ASM medium and as control LB medium were inoculated with indicated strains aerobically for 24 h at 37°C. An OD 578 of 0.5 was used for the inoculation. Afterwards, 1*10^5 ^phages were added which describes the initial phage concentration. After incubation for additional 24 h at 37°C the colony forming units (CFU) as well as the plaque forming units (PFU) were determined. To determine the change in phage concentration we divided the final phage concentration after 24 h by the initial phage concentration. To determine the effect of alginate the same experiment was performed in LB with purified alginate using increasing concentrations in a range of 50 *μ*g/ml to 1 mg/ml. Alginate was purified from mucoid *P. aeruginosa *strain FRD1 [34] as described previously [36].

## Authors' contributions

JG participated in the design of the study, isolated and characterized the phages, annotated the genome, performed host specificity observations of clinical isolates as well as the ASM assay and drafted the manuscript. AW provided the ASM medium and participated in the ASM assay. BB assisted with bioinformatic analyses. MK, KS, CR and JS were involved in the host specificity study of the 100 environmental strains which were provided and investigated by KS and JS. Electron microscopically examinations were done by MR. DJ contributed to the design of the study. MS designed the study, carried out bioinformatic analysis and revised the manuscript. All authors read and approved the final manuscript.

## Supplementary Material

Additional file 1 **Supplementary Figure S1**. Graph and schematic representation of a Mauve comparison using phage JG024, phage PB1 and SN. Click here for file
