# Supplementary material for: Characterization of JG024, a pseudomonas aeruginosa PB1-like broad host range phage under simulated infection conditions
Source: BMC Microbiol. 2010 Nov 26;10:301. doi: 10.1186/1471-2180-10-301 (PMC3008698; doi:10.1186/1471-2180-10-301)
Supplement: Additional file 1 — Supplementary Figure S1. Graph and schematic representation of a Mauve comparison using phage JG024, phage PB1 and SN. [file 1471-2180-10-301-S1.PDF]

Figure S1

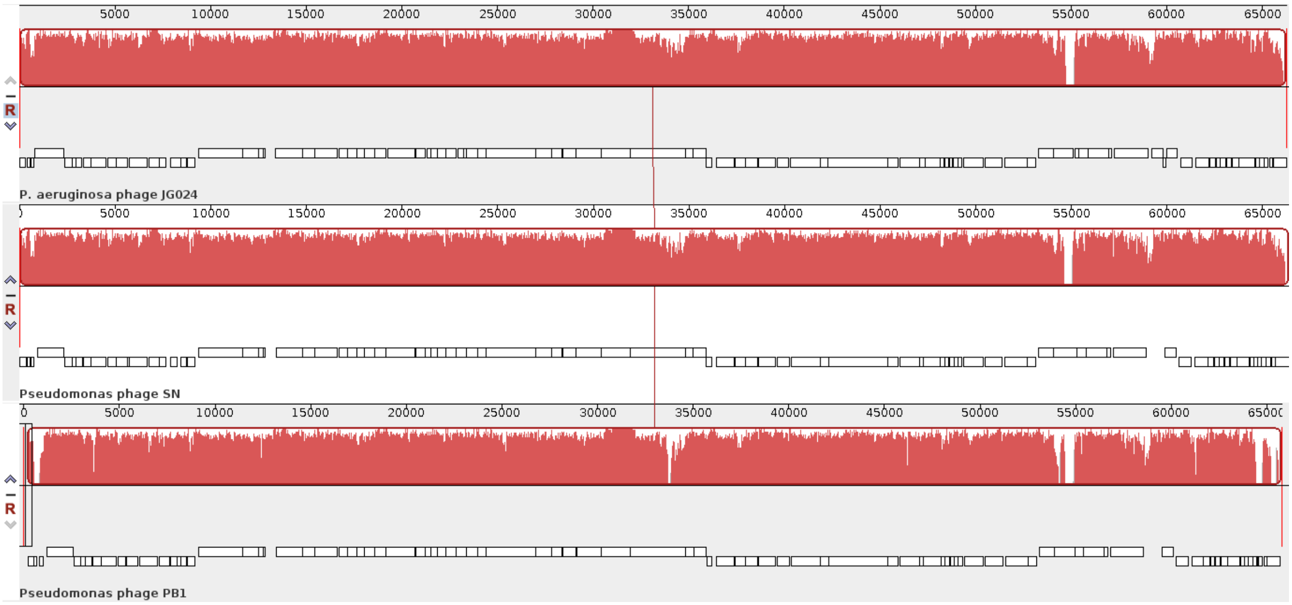

Legend Figure S1:

Graph and schematic representation of a Mauve comparison using phage JG024, phage PB1 and SN (from top to bottom). The linear genome sequences of each phage, the position of the individual Orfs and their orientation are indicated. A ruler displaying the base count in kb is on top of the respective phage genome. The red graph indicates the mutual conservation of the respective DNA sequence. A low red graph indicates low conservation. A red graph of zero indicates genome areas with insertions or deletions.
